# Evaluation of Biological and Functional Changes in Healthy Smokers Switching to the Tobacco Heating System 2.2 Versus Continued Tobacco Smoking: Protocol for a Randomized, Controlled, Multicenter Study

**DOI:** 10.2196/11294

**Published:** 2018-08-24

**Authors:** S Michael Ansari, Nicola Lama, Nicolas Blanc, Marija Bosilkovska, Andrea Donelli, Patrick Picavet, Gizelle Baker, Christelle Haziza, Frank Lüdicke

**Affiliations:** ^1^ Philip Morris International Research & Development Neuchâtel Switzerland

**Keywords:** smoking, tobacco, harm reduction, tobacco products, risk, heated tobacco, smoking cessation, biomarkers, metabolic networks, pathways

## Abstract

**Background:**

Tobacco harm reduction, substituting less harmful tobacco products for combustible cigarettes, is a complementary approach for smokers who would otherwise continue to smoke. The Philip Morris International (PMI) Tobacco Heating System (THS) 2.2 is a novel tobacco product with the potential to reduce the risk of harm in smokers compared to continued smoking of combustible cigarettes. It heats tobacco electrically in a controlled manner, never allowing the temperature to exceed 350°C, thereby preventing the combustion process from taking place and producing substantially lower levels of toxicants while providing nicotine, taste, ritual, and a sensory experience that closely parallels combustible cigarettes. Previous clinical studies have demonstrated reduced exposure to the toxicants (approaching the levels observed after quitting) for smokers who switched to THS 2.2, for three months. For adult smokers who would otherwise continue smoking combustible cigarettes, switching to THS 2.2 may represent an alternative way to reduce the risk of tobacco-related diseases.

**Objective:**

This study aimed to further substantiate the harm reduction potential of THS 2.2 by demonstrating favorable changes in a set of 8 coprimary endpoints, representative of pathomechanistic pathways (ie, inflammation, oxidative stress, lipid metabolism, respiratory function, and genotoxicity), linked to smoking-related diseases, in smokers switching from combustible cigarettes to THS 2.2.

**Methods:**

This study was a randomized, controlled, two-arm parallel group, multicenter ambulatory US study conducted in healthy adult smokers switching from combustible cigarettes to THS 2.2 compared with smokers continuing to smoke combustible cigarettes for six months. Subjects had a smoking history of at least ten years and did not intend to quit within the next six months.

**Results:**

Enrollment started in March 2015 and the trial was completed in September 2016. In total, 984 subjects were randomized (combustible cigarettes, n=483; THS 2.2, n=477), and 803 completed the study. The results are expected to be available in a subsequent publication in 2019.

**Conclusions:**

In this paper, we describe the rationale and design for this clinical study that focused on the evaluation of THS 2.2’s potential to reduce the risk of smoking-related diseases compared with that of combustible cigarettes. This study will provide insights regarding favorable changes in biological and functional endpoints informed by effects known to be seen upon smoking cessation.

**Trial Registration:**

ClinicalTrials.gov NCT02396381; http://clinicaltrials.gov/ct2/show/NCT02396381 (Archived by WebCite at http://www.webcitation.org/71PCRdagP)

**Registered Report Identifier:**

RR1-10.2196/11294

## Introduction

Cigarette smoking is the leading cause of preventable disease in the US, accounting for more than 480,000 smoking-related deaths every year. More than 16 million Americans live with a smoking-related disease [1]. Although the smoking prevalence in the US has declined from 21% to 17% over the last decade, an estimated 40 million people currently smoke cigarettes in the US [2], and one billion people worldwide continue to smoke [3]. Smoking is addictive, and smoking cessation is difficult for many smokers, even though it is the best way to reduce the risk of developing smoking-related diseases.

In addition to the prevention of smoking initiation and the promotion of smoking cessation, tobacco harm reduction is being recognized as a valuable and promising approach to further accelerate the decline in smoking prevalence and smoking-related population harm [4]. Tobacco harm reduction is based on switching smokers to markedly less harmful alternative products, referred to by the Food and Drug Administration as modified risk tobacco products (MRTP). The US Family Smoking Prevention and Tobacco Control Act defines an MRTP as “any tobacco product that is sold or distributed for use to reduce harm or the risk of tobacco-related disease associated with commercially marketed tobacco products” [5].

Importantly, to improve health at the population level, these substitutes for cigarettes must be acceptable for smokers, providing adequate nicotine delivery and satisfaction to prevent relapse to cigarette smoking. In this context, Philip Morris International (PMI) has developed Tobacco Heating System (THS) 2.2, as a candidate MRTP that has been designed to provide nicotine to smokers, who otherwise would have continued to smoke, offering sensory and ritual aspects like cigarettes while reducing the exposure to harmful and potentially harmful constituents (HPHC) found in cigarette smoke [6-8]. Additional studies have been conducted on alternatives to cigarettes. A carbon heated tobacco product developed by PMI has demonstrated markedly reduced biomarkers of exposure to HPHCs (NCT02503254) [9]. British American Tobacco has developed a product that heats rather than burns tobacco, significantly reducing exposure to smoke toxicants to levels comparable to quitting tobacco [10]. Japan Tobacco has also introduced a smokeless tobacco product with data reflecting substantially lower exposure to smoke toxicants [11]. Electronic cigarettes have also demonstrated reduced exposure to smoke toxicants compared to cigarettes [12].

THS 2.2 uses a precisely controlled heating device into which a specially designed tobacco product, the Tobacco Stick, is inserted and heated to generate an aerosol. The THS 2.2 heater starts heating the Tobacco Stick in a controlled and gradual manner, with the temperature set between 320°C and 350°C. Heating the Tobacco Stick in a controlled manner and not allowing the temperature to exceed 350°C prevents the combustion process from taking place. The elimination of combustion results in a significant reduction in the production and exposure to HPHCs [13] while the nicotine is delivered to the THS 2.2 user in a way that is like cigarettes. The holder must be recharged after each use, and the charger must be recharged after approximately 20 uses.

PMI has designed a multilayered scientific program to assess whether THS 2.2 can significantly reduce the risk of harm and smoking-related diseases in adult smokers who otherwise would have continued to smoke cigarettes. Preclinical and clinical studies have been conducted on THS 2.2 and its predecessors. Aerosol from THS 2.2 contains, on average, approximately 90% less HPHCs found in smoke from a standard reference cigarette, which translates to a reduced toxicity of approximately 90% [13]. Chronic exposure to THS 2.2 aerosols in animal models, even at high concentrations, resulted in lower systemic toxicity, with reduced lung inflammation and histopathological changes in the nasal epithelium and lung tissue [14]. Furthermore, in the ApoE^−/−^ mouse model, which is commonly used to study atherosclerosis and emphysema, exposure to THS 2.2 aerosol did not induce a change in the lipid profile or enlargements of aortic plaque area, nor lung inflammation or emphysema, unlike cigarette smoke. Additionally, switching from cigarette smoke to THS 2.2 aerosol exposure reversed inflammation, and halted aortic plaque growth and the progression of emphysema in a manner that mimics smoking cessation [15].

In humans, previous clinical studies showed a similar nicotine absorption profile in smokers using a single Tobacco Stick or smoking a cigarette [16] and demonstrated reductions in the levels of 15 biomarkers of exposure to HPHCs in healthy adult smokers who switched exclusively to THS 2.2 for five days in confinement or for three months in ambulatory setting relative to cigarettes. The magnitude of reductions was comparable to what was observed in adult smokers who abstained from smoking [16-18].

In summary, the available clinical evidence demonstrates that humans who switch from cigarettes to THS 2.2, are exposed to significantly lower levels of selected HPHCs. This observed reduction is of a similar magnitude as that observed in smokers who abstain from smoking, which has been referred to as the “gold standard” for the assessment of candidate MRTPs [19,20]. Considering the preclinical and clinical data on exposure reduction, it is reasonable to assume that the reduction in exposure to toxicants leads to favorable changes in biological and functional endpoints involved in smoking-related disease development and progression.

Smoking-related diseases have a complex etiology and involve several mechanisms that affect multiple organ systems [20]. Chronic exposure causes alterations at the cellular and tissue level that result in physiological changes and disrupt multiple biological processes, contributing to disease manifestation. Oxidative stress and inflammation play a critical role in the development and progression of the major smoking-related diseases: cardiovascular disease, chronic obstructive pulmonary disease, and cancer. There is no single clinical risk endpoint (CRE) or biomarker that is an adequate surrogate measure for the multiple adverse health effects associated with smoking, and that can fully demonstrate a reduction in risk.

Because smoking-related diseases often take decades to manifest, conducting long-term epidemiological studies would require decades to demonstrate the reduced risk of THS 2.2.

Thus, the demonstration of favorable changes in a set of CREs that are representative of multiple biological processes, physiological systems, and mechanistic pathways in smokers who switch to THS 2.2 is a reasonable approach to provide scientific evidence in a pre-market setting that THS 2.2 can reduce the risk of harm and smoking-related diseases.

This study will assess the risk profile of THS 2.2 in a pre-market setting and support risk assessment of this novel tobacco product together with all available evidence as one set of logical, empirically coherent, and consistent data.

## Methods

### Study Design

**Figure 1:**
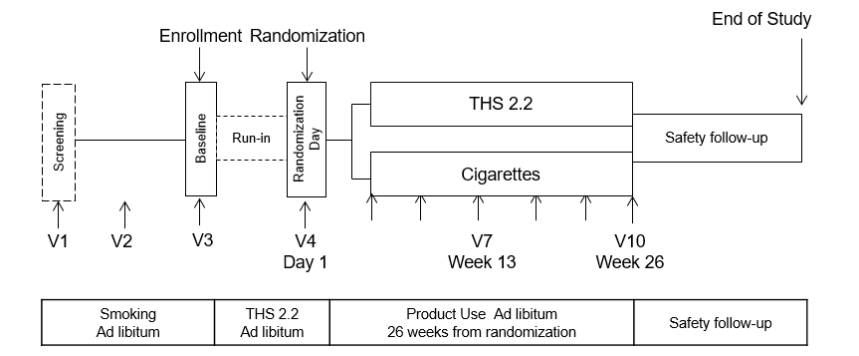
Design of the study. Eligible subjects were provided with the Tobacco Heating System 2.2 (THS 2.2) at visit 3 (V3) and were allowed to use the product freely during the 6- to 10-day run-in period until V4. Those willing to use THS 2.2 exclusively during the study were randomized to the THS 2.2 or cigarette arms.

This study was a randomized, controlled, two-arm parallel group, multicenter study comparing multiple CREs in smokers switching from cigarettes to THS 2.2 and smokers continuing to smoke cigarettes for six months (NCT02396381). This open-label study was conducted at 20 clinical research centers in the US. The study design is illustrated in Figure 1. The first subject was screened on March 12, 2015, and the last subject completed the study on September 13, 2016.

After visit 1 (V1), the screening visit, during which eligibility criteria were checked, participants received study supplies, such as a container for urine collection and an electronic diary. Participants were trained by the site staff on how to collect 24-hour urine and how to fill the diary daily. Urine collection started in the morning of the day preceding a study visit and ended 24 hours later on the morning of a visit. Starting from V2, subjects recorded all nicotine and tobacco-containing products used in their daily diary. At V3, after a recheck of selected eligibility criteria, participants were enrolled in the study, and baseline assessments were performed, including blood and 24-hour urine sample collection for biomarker analysis. After enrollment, at the end of V3, THS 2.2 units were distributed to all participants to be used during an eight-day run-in period to get familiar with the use of THS 2.2. The use of other tobacco and nicotine products was also permitted.

At the end of the run-in period (V4), all enrolled participants willing to use THS 2.2 for the next six months were randomized in a 1:1 ratio to either the THS 2.2 or the combustible cigarette (CC) arms. The sponsor provided THS 2.2, and participants were instructed to use it ad libitum. Participants randomized to the CC arm were asked to purchase and smoke their own brand of cigarettes ad libitum. Randomization was performed using an interactive voice and web response system using gender and study site as stratification criteria. Use of tobacco or nicotine products other than the allocated product during the randomized exposure period did not lead to the removal of the participant from the study. For participants randomized to the CC arm, the use of THS 2.2 was not allowed. Therefore, the THS 2.2 device and remaining Tobacco Sticks were collected from subjects randomized to the CC arm after the run-in period.

Subjects returned to the clinic each month for safety checks and resupply of THS 2.2 Tobacco Sticks when needed. Major study visits occurred every three months (V7, V10) for lung function assessment and collection of blood and 24-hour urine for biomarker analysis. After V10 (six months postrandomization), subjects who completed the study were invited to participate in an extension study for an additional six months (NCT02649556). Subjects participating in the extension study continued to use the same product to which they had been assigned and continued to visit the same clinical sites monthly. The purpose of the extension study was to follow the study participants for a more extended period and to further describe changes in CREs, lung function, as well as biomarkers of exposure. For that purpose, blood and 24-hour urine samples were collected, and lung function was assessed at V16 (month 12).

Subjects choosing not to enroll in the extension study entered into a 28-day safety follow-up period. Adverse events were recorded from the signature of informed consent onwards until the end of the safety follow-up period.

### Objectives and Endpoints of the Study

The smokers’ health profile (Table 1) is a collection of 8 co-primary CREs that together cover various mechanistic and pathological pathways (ie, inflammation, oxidative stress, lipid metabolism, changes in respiratory function, and genotoxicity), that are known to contribute to smoking-related diseases, such as cardiovascular and respiratory disease as well as cancer [20]. These CREs, selected for the smokers’ health profile, are also known to be reversible upon smoking cessation within a few days to one year. The individual CREs were chosen for (1) their link to smoking-related diseases, (2) evidence that the smoking status influences the CREs, and (3) their favorable change upon smoking cessation within a timeframe feasible for the study duration (Table 1). Many of these CREs are mentioned in the 2010 Surgeon General's Report entitled *How Tobacco Smoke Causes Disease: The Biology and Behavioral Basis for Smoking-Attributable Disease* [20].

Biomarkers of exposure to carbon monoxide (CO) and 4-(methylnitrosamino)-1-(3-pyridyl)-1-butanone (nicotine-derived nitrosamine ketone; NNK) were included in the list of CREs, as they contribute to exacerbate acute ischemic effects (ie, CO), and are known tobacco-specific carcinogenic compounds (ie, NNK).

The primary objective of the study was to indicate favorable changes in the 8 CREs in the smokers’ health profile. The evaluation criteria for the study was that at least five out of the eight CREs would show statistically significant favorable changes.

The secondary objectives and their related CREs included additional biological and functional CREs supportive of the smokers’ health profile, biomarkers of exposure to HPHCs, assessments of subjective effects, and safety CREs (Table 2).

**Table 1:** Components of the smokers’ health profile.

Component	Related physiological process	Sample	Expected change in THS 2.2^a^ arm	Expected timeframe of reversibility
High-density lipoprotein cholesterol	Lipid metabolism	Serum	Increase	3 months [21]
White blood cell count	Inflammation	Blood	Decrease	6-12 months [21]
Soluble intercellular adhesion molecule-1	Endothelial dysfunction	Serum	Decrease	4 weeks [22,23]
11-dehydrothromboxane B2	Platelet activation	Urine	Decrease	2-4 weeks [24,25]
8-epi-prostaglandin F2alpha	Oxidative stress	Urine	Decrease	1-2 weeks [26,27]
Carboxyhemoglobin	Transport of oxygen by hemoglobin	Blood	Decrease	1-7 days [28]
Forced expiratory volume in one second	Lung function	None	Increase	6-12 months [29-31]
Total 4-(methylnitrosamino)-1-(3-pyridyl)-1-butanol	Exposure to carcinogenic potentially harmful constituents	Urine	Decrease	3 months [32]

^a^THS 2.2: Tobacco Heating System 2.2.

The selection criteria for the biomarkers of exposure included: (1) detectability, reproducibility, and precision of the analytical methods, (2) specificity for the toxic exposure or reliable surrogate of exposure to HPHCs, (3) presence in the gas or particulate phases, (4) formation at different temperatures, and (5) relation to different chemical and organ toxicity classes. Additionally, the relationship between levels of urinary biomarkers of exposure and nicotine equivalents were assessed at six months, to evaluate the effect of combined product use (cigarettes and THS 2.2 Tobacco Sticks) on the smokers’ health profile, the intention to use THS 2.2, and the change in tobacco dependence in smokers switching from cigarettes to THS 2.2.

### Study Measurements

The details of the assessments performed during the study are provided in the schedule of events (Multimedia Appendix 1) [33]. Subjects’ reported smoking intensity (cigarettes per day during the previous year) and duration, as well as subjects’ lifestyle characteristics (diet, alcohol intake, exercise, sleep deficit, living in a household with other smokers), were collected using questionnaires at baseline. Standard spirometry was conducted pre- and postbronchodilator (salbutamol), and lung volumes were assessed using the Helium Dilution Technique. Both CREs were assessed following the respective guidelines of the European Respiratory Society [34,35]. Full lung function assessment was read centrally. Blood collection and urine sampling from the 24-hour urine were conducted for CREs and biomarkers of exposure analyses at baseline (V3), at month 3 (V7), and at month 6 (V10). One central laboratory was responsible for storage and shipment of urine and blood samples, and multiple laboratories performed the analyses using validated methods to assess all laboratory safety parameters, biomarkers of exposure, and CREs (Multimedia Appendix 2) [36].

**Table 2:** Secondary objectives and endpoints of the study.

Objective	Endpoint
To evaluate self-reported product use over the duration of the study	Number of cigarettes or THS 2.2^a^ Tobacco Sticks used daily, as reported in the product use electronic diary
To determine short-term changes of the smokers’ health profile at month 3	All components of the smokers’ health profile
To indicate the reduction of exposure to HPHC^b^ at month 3 and month 6	Biomarkers of exposure to carbon monoxide (CO): CO in exhaled breath Biomarker of exposure to 1,3-butadiene: monohydroxybutenylmercapturic acid in urine Biomarker of exposure to acrolein: 3-hydroxypropylmercapturic acid in urine Biomarker of exposure to N-nitrosonornicotine: total N-nitrosonornicotine in urine Biomarker of exposure to acrylonitrile: 2-cyanoethylmercapturic acid in urine Biomarker of exposure to benzo[a]pyrene: 3-hydroxybenzo[a]pyrene in urine Biomarker of exposure to crotonaldehyde: 3-hydroxy-1-methylpropylmercapturic acid in urine Biomarker of exposure to pyrene: total 1-hydroxypyrene in urine
To describe the levels of nicotine exposure at month 3 and month 6	Nicotine equivalent: molar sum of free nicotine, nicotine-glucuronide, free cotinine, cotinine-glucuronide, free trans-3′-hydroxycotinine, trans-3′-hydroxycotinine-glucuronide in urine Nicotine and cotinine in plasma
To describe the changes of clinical risk endpoints associated with respiratory diseases, cardiovascular diseases, and xenobiotics	Lung function (spirometry postbronchodilator): FEV_1_^c^, FVC^d^, FEV_1_/FVC, FEF^e^ 25-75 Lung volumes (lung volume prebronchodilator): functional residual capacity, vital capacity, total lung capacity, inspiratory capacity, and residual volume at month 3 and month 6 Cough symptoms (intensity and frequency), amount of sputum production, and bothersomeness of cough symptoms, from the cough questionnaire at month 3 and month 6 Lung function (spirometry prebronchodilator): FEV_1_, FVC, FEV_1_/FVC, FEF 25-75 at month 6 Lung function (spirometry, pre- and postbronchodilator): bronchodilator reversibility in FEV_1_ at month 6 Myeloperoxidase, apolipoprotein A1 and B, low-density lipoprotein cholesterol, and high-sensitivity C-reactive protein in serum at month 3 and month 6 Fibrinogen and homocysteine in plasma at month 3 and month 6 Platelet count and hemoglobin glycosylated in whole blood at month 3 and month 6 Albumin in urine at month 3 and month 6 Blood pressure, weight, and waist circumference at month 3 and month 6 Cytochrome P450 2A6 activity in plasma at month 3 and month 6
To describe the changes in subjective effects of smoking at month 3 and month 6	Product evaluation (Modified Cigarette Evaluation Questionnaire) [37]
To evaluate the safety profiles associated with THS 2.2 and cigarettes over the course of the study	Adverse Events, Serious Adverse Events, and device events, including THS 2.2 malfunction or misuse Vital signs, body weight, and body mass index Respiratory symptoms Spirometry Electrocardiogram Clinical chemistry, hematology, and urine analysis safety panel Physical examination Concomitant medications

^a^THS 2.2: Tobacco Heating System 2.2.

^b^HPHC: harmful and potentially harmful constituents.

^c^FEV_1_: forced expiratory volume in one second.

^d^FVC: forced vital capacity.

^e^FEF: forced expiratory flow.

Collection of 24-hour urine started at the subject’s home on the morning of the day before the scheduled visit and ended the morning of the day of the study visit. Blood was collected after at least 10 hours of fasting except for carboxyhemoglobin measurement. Exhaled breath was measured for CO using a Smokerlyzer device (Bedfont Scientific Ltd, UK) as another biomarker of exposure to CO. Cough assessment by visual analog scale and Likert scales (intensity of a cough, frequency of a cough, and the amount of sputum collection) were conducted at baseline (V3), at month three, and at month six.

### Enrollment

This study enrolled current adult smokers of nonmenthol cigarettes who did not intend to quit smoking within the next six months. At least 950 participants were to be randomized. Once 950 subjects had been randomized, no additional subjects were enrolled; however, all subjects who were already enrolled and started the run-in period were still randomized.

The main inclusion and exclusion criteria are listed in Textbox 1. Participants had at least ten years of smoking history and smoked at least ten cigarettes per day over the last 12 months based on self-reporting. There were no limitations on race or ethnicity other than a quota on Caucasian subjects to ensure that they did not represent more than 75% of randomized subjects. Participants of each gender were limited to no more than 60% of the study population.

Approval for the study was granted by one central Institutional Review Board for each of the participating sites. All participants provided written informed consent before the start of the study. The study was conducted following Good Clinical Practice guidelines and the ethical principles of the Declaration of Helsinki [38-40]. Study participants were remunerated for the time they devoted to the study in line with the local market practice and approved by the Institutional Review Board.

### Statistical Considerations

A sample size of 950 subjects (randomized 1:1) was calculated to be enough to attain a statistical power of >99% to show statistically significant favorable changes in at least five out of eight CREs of the smokers’ health profile at six months. The Hailperin-Rüger approach will be used to adjust for test multiplicity [41,42].

Although this was an open-label study, and the subjects and the investigators or their designees were unblinded to the subject’s study arm after randomization, a limited degree of blinding was implemented during the conduct of the study, including the data review and data analysis process. The study statisticians and clinical scientists involved with the definition of the analyses were blinded to the actual values of primary CREs from the time of randomization until database lock.

The primary analysis will be run on the full analysis set (FAS) of subjects based on their actual product exposure [43] according to predefined product use pattern categories (Table 3). Subjects switching to THS 2.2 and those smoking cigarettes will be identified by THS‑use and CC‑use product use categories, respectively. Results of the Dual-use versus CC-use comparison will also be evaluated in secondary and exploratory analysis tables. Only subjects with at least one record of reported product use postrandomization will be included in the primary analysis. Missing data will be considered as missing at random, and each CRE will be analyzed using a mixed-effect model repeated measure adjusting for value of the CRE at baseline and its interaction with visit, gender, Caucasian origin, product use pattern category, and other lifestyle covariates relevant for each CRE following examination of baseline comparability between THS-use and CC-use (see Table 4). Site will be included as a random effect. Sensitivity analyses will be conducted for the FAS considering alternative missing imputation approaches for primary endpoints and product use. Sensitivity analysis will also be conducted on the FAS by randomization arm.

**Main criteria for inclusion and exclusion of subjects.**

**Inclusion criteria**

- Healthy smoker
- At least 30 years old
- Smoking history of at least 10 years
- Smoking history of at least 10 nonmenthol cigarettes per day on average in the 12 months preceding the screening
- No intention to quit smoking within the next six months

**Exclusion criteria**

- Clinically relevant gastrointestinal, renal, hepatic, neurological, hematological, endocrine, oncological, urological, pulmonary, immunological, psychiatric, or cardiovascular disorders or any other conditions that would jeopardize the safety of the participant or affect the validity of the study results
- Abnormal findings on physical examination, in the medical history, or in clinical laboratory results deemed clinically relevant by investigators (as per the common terminology criteria for adverse events)
- Acute illness (eg, upper respiratory tract infection, viral infection) requiring treatment within 30 days before enrollment in the study
- Use of any prescribed or over-the-counter systemic medications with an impact on the clinical risk endpoints of the smokers’ health profile within five half-lives of the medication before study enrollment, except over-the-counter vitamin supplements, hormonal contraceptives, and hormone replacement therapy
- FEV_1_/FVC below 0.7 and FEV_1_ below 80% predicted value at postbronchodilator spirometry (FEV_1_ refers to the forced expiratory volume in 1 second while FVC refers to forced vital capacity)
- Pregnancy or breastfeeding
- Unwilling to use an acceptable method of effective contraception (females only)

**Table 3:** Principal categories of actual product use pattern.

Category label	Definition
THS^a^‑use	≥1 THS 2.2 Tobacco Sticks or cigarettes and ≥70% THS 2.2 Tobacco Stick use over the entire analysis period and ≥70% THS 2.2 Tobacco Stick use on ≥50% of the days in the analysis period
Dual‑use	≥1 THS 2.2 Tobacco Sticks or cigarettes and 1%≤ THS 2.2 Tobacco Sticks <70% over the entire analysis period or THS 2.2-use and CC^b^-use do not apply to <50% of these days
CC‑use	≥1 THS 2.2 Tobacco Sticks or cigarettes and <1% THS 2.2 Tobacco Sticks over the entire analysis period, and <1% THS 2.2 Tobacco Sticks on ≥50% of the days in the analysis period
Other use	General category encompassing subjects with missing product use, subjects using e‑cigarettes or other tobacco products, subjects who quit, or subjects who switched across different use patterns between consecutive analysis periods

^a^THS: Tobacco Heating System.

^b^CC: combustible cigarette.

**Table 4:** Baseline covariates for the analysis of primary endpoints.

Endpoint	Defined covariates^a^	Evaluated covariates^a^
High-density lipoprotein cholesterol	Age, smoking intensity	Smoking duration, diet, alcohol intake, exercise, body mass index
Total white blood cell	Age, smoking intensity	Smoking duration, race/ethnicity, sleep deficit
sICAM-1^b^	Age, smoking intensity	Smoking duration
11‑DTX-B2^c^	Age, smoking intensity	Living in household with smokers
8‑epi-PGF2α^d^	Age, smoking intensity	Smoking duration, body mass index, weight
Carboxyhemoglobin	Age, smoking intensity	Living in household with smokers
FEV_1_^e^	Smoking intensity	Sex, age^f^, race and ethnicity, height, diet, exercise, body mass index, weight, smoking duration
Total NNAL^g^	Age, smoking intensity	Living in household with smokers

^a^The model will include terms for the “Defined Covariates” and for the subset of “Evaluated Covariates” selected if found to be significant at 10% level (between THS-use and CC-use) at baseline.

^b^sICAM-1: soluble intercellular adhesion molecule 1.

^c^11-DTX-B2: 11-dehydrothromboxane B2.

^d^8-epi-PGF2α: 8-epi-prostaglandin F2alpha.

^e^FEV_1_: forced expiratory volume in 1 second and measured as percent of predicted value.

^f^Age is not included in the defined covariates because it is accounted for in the percent predicted assessment.

^g^NNAL: 4-(methylnitrosamino)-1-(3 pyridyl)-1-butanol.

For the primary analysis, substantiation that switching to THS 2.2 from cigarettes modifies the risk of smoking-related diseases will rely on the following criteria. First, all CREs in the smokers’ health profile must shift in the same direction as they would upon smoking cessation. Second, switching to THS 2.2 must show a statistically significant improvement in at least five of the 8 components of the smokers’ health profile, with each CRE evaluated using a one-sided alpha of 1.5625%, corresponding to half of the Hailperin-Rüger adjusted type I error (.031). The Hailperin-Rüger approach calculates the statistical significance that is required for each test when at least five of the 8 primary CREs in the smokers’ health profiles are required to be significant to maintain the overall study-wise alpha-level of 5%. Effect estimates will be presented accompanied by 2-sided 96.875% (100-alpha %) confidence intervals. It will be finally evaluated if most of the effect of smoking cessation is preserved in subjects switching to THS 2.2, based on the results of an integrated analysis pooling data from a separate smoking cessation study (NCT02432729) designed to benchmark the clinical, biological, and functional changes in smokers who are continuously abstinent from smoking for 1 year. The methods and results will be reported in a separate manuscript. There were no interim analyses planned.

**Figure 2:**
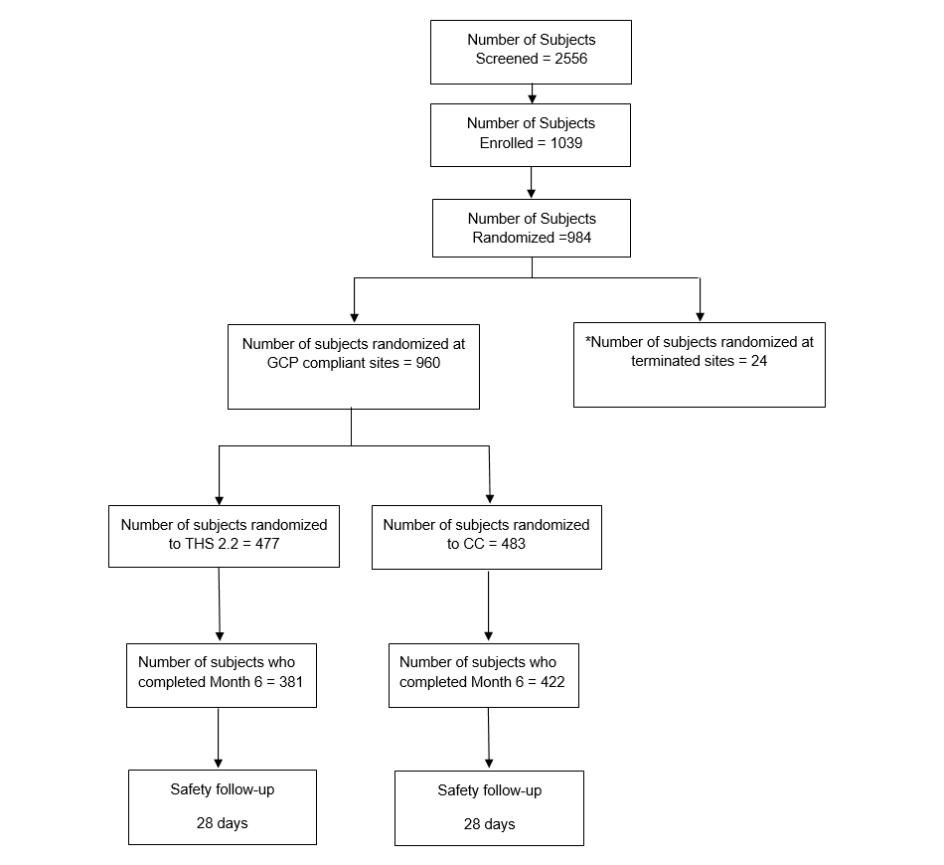
Flow chart of study participants. Asterisk indicates sites terminated due to noncompliance with Good Clinical Practice (GCP). CC: combustible cigarette; THS 2.2: Tobacco Heating System 2.2.

### Participants

For the study, 2,556 subjects were screened, 1,039 were enrolled, 984 were randomized (483 to CC arm and 477 to THS 2.2 arm), and 803 completed the study (Figure 2). The database lock of the study is completed.

## Results

Enrollment started in March 2015 and the trial was completed in September 2016. The results of this paper are expected in 2019.

## Discussion

### Preliminary Insights

The diseases attributed to smoking are complex. Continuous exposure to HPHCs affects multiple organ systems, disease pathways, and mechanisms, such as inflammation, oxidative stress, platelet activation, and lipid metabolism, which coincide, leading gradually to the development of smoking-related diseases over the course of years. This study examined changes in biological and functional CREs in adult smokers switching to THS 2.2 in an ambulatory, near real-life setting. Because no single CRE is validated as a surrogate measure for any smoking-related disease, the primary endpoint of this study was a selection of equally important, nonhierarchical, co-primary CREs defined as the smokers’ health profile.

The analysis of this study uses a robust approach in the field of tobacco harm reduction. All co-primary CREs of the smokers’ health profile must shift in the same direction as they would upon smoking cessation, and at least five of the 8 components of the smokers’ health profile must be significantly improved statistically in THS 2.2 users compared with those who continued smoking cigarettes. Significance will be evaluated using a one-sided test with the Hailperin-Rüger adjusted alpha-level.

Furthermore, the planned analysis approach considers baseline comparability of confounding factors that can potentially influence study results, such as exercise, diet, alcohol intake, and potential exposure to passive smoking. Additional CREs that are representative of various mechanistic or pathological pathways will be evaluated in the secondary objectives to support the analysis of the primary objective. Because smoking alters multiple pathways, tissues, and organs, which together contribute to disease risk, this approach will provide coherent and multifaceted scientific evidence of the reduced-risk potential of THS 2.2.

The ambulatory setting will provide information not only on product consumption and combined or dual-use (smoking of cigarettes in addition to using THS 2.2) but also on user satisfaction and acceptance of the product, as assessed by the proportion of product used, the “intent to use” questionnaire, and the modified cigarette evaluation questionnaire.

In summary, results from this study will be a noteworthy addition to the growing body of data from the assessment program to scientifically substantiate that THS 2.2 can potentially reduce the risk of smoking-related diseases [6]. The design and approach used in the present study should be considered in light of its limitations. One potential limitation is that the study population might not match the general population of potential THS 2.2 consumers. The study enrolled only smokers who smoked at least 10 cigarettes a day. Also, the study may provide only limited insight on the effect of THS 2.2 in various races and ethnicities.

### Conclusions

This study is part of a multilayered assessment program designed to evaluate whether THS 2.2 can potentially reduce the risk of smoking-related diseases relative to continued smoking. The results of this study will confirm whether the reduction in exposure to HPHCs when switching from cigarettes to THS 2.2 leads to statistically significant favorable changes in CREs linked to smoking disease and following the direction expected upon smoking cessation. Detailed information on product use, product satisfaction, and acceptance will also emerge from this study. This study will provide evidence to substantiate the reduced-risk potential of THS 2.2. Longer term duration of exposure is needed to evaluate these changes in biological and functional biomarkers further.

## Appendix

Multimedia Appendix 1 Schedule of events.

Multimedia Appendix 2 Methods and measurements.

## Abbreviations

8-epi-PGF2α: 8-epi-prostaglandin F2alpha
11-DTX-B2: 11-dehydrothromboxane B2
CC: combustible cigarette
CO: carbon monoxide
CRE: clinical risk endpoint
FAS: full analysis set
FEF: forced expiratory force
FEV _1_: forced expiratory volume in 1 second
FVC: forced vital capacity
HPHC: harmful and potentially harmful constituents
PMI: Philip Morris International
MCEQ: Modified Cigarette Evaluation Questionnaire
MRTP: modified risk tobacco product
NNAL: 4-(methylnitrosamino)-1-(3 pyridyl)-1-butanol
NNK: nicotine-derived nitrosamine ketone
sICAM-1: soluble intercellular adhesion molecule 1
THS 2.2: Tobacco Heating System 2.2
V: visit
