# Supplementary material for: Evaluation of Biological and Functional Changes in Healthy Smokers Switching to the Tobacco Heating System 2.2 Versus Continued Tobacco Smoking: Protocol for a Randomized, Controlled, Multicenter Study
Source: JMIR Res Protoc. 2018 Aug 24;7(8):e11294. doi: 10.2196/11294 (PMC6128954; doi:10.2196/11294)
Supplement: Multimedia Appendix 1 [file resprot_v7i8e11294_app1.pdf]

Table 3. Schedule of events.

Visits<br>Assessments	Screening	Distribution of containers Diary	Enrollment Baseline Start of Run-in Period	End of run-in period Randomization	Exposure ambulatory period						Safety follow-up [m]
	V1	V2	V3	V4	V5	V6	V7	V8	V9	V10	28 days
Study week (W)<br>Study day (D)	–	–	–	Day 1	W4	W9	W13	W17	W22	W26	–
Informed consent and additional ICFs for biobanking	●	–	–	–	–	–	–	–	–	–	–
Information on the risk of smoking, advice on smoking cessation, and debriefing on THS 2.2	●	–	●	●	●	●	●	●	●	●	–
Demonstration of THS 2.2	●	–	–	–	–	–	–	–	–	–	–
Inclusion/exclusion criteria	●	–	●	–	–	–	–	–	–	–	–
Enrollment	–	–	●	–	–	–	–	–	–	–	–
Randomization	–	–	–	●	–	–	–	–	–	–	–
Prochaska questionnaire [a]	●	–	–	–	–	–	–	–	–	–	–
Readiness to comply with study procedures and to use THS 2.2 for 26 weeks	●	–	–	●	–	–	–	–	–	–	–
Smoking questionnaire	●	–	–	–	–	–	–	–	–	–	–
Baseline lifestyle assessment	–	–	●	–	–	–	–	–	–	–	–
Demographics, medical history, prior medication	●	–	–	–	–	–	–	–	–	–	–
Concomitant medication	●	●	●	●	●	●	●	●	●	●	●
Concomitant diseases	●	–	–	–	–	–	–	–	–	–	–
B: HIV, hepatitis B and C	●	–	–	–	–	–		–	–	–	–
U: Drug screen/cotinine screen	●	–	–	–	–	–	–	–	–	–	–
Alcohol test	●	–	–	–	–	–	–	–	–	–	–
Chest X ray [b]	●	–	–	–	–	–	–	–	–	–	–
U: Pregnancy test (females)	●	–	●	–	●	●	●	●	●	●	–
B/U: Clinical chemistry, hematology, urine analysis [c]	●	–	●	–	–	–	●	–	–	●	–
ECG	●	–	–	–	–	–	–	–	–	●	–
Vital signs [d]	●	–	●	●	●	●	●	●	●	●	–
Waist circumference	–	–	●	–	–	–	●	–	–	●	–
Height, weight and BMI [e]	●	–	●	–	–	–	●	–	–	●	–
Physical examination	●	–	●	–	–	–	●	–	–	●	–
Dispensing of THS 2.2 [f]	–	–	●	●	●	●	●	●	●	●	–
CO breath test [g]	–	–	●	–	–	–	●	–	–	●	–
U: BoExp	–	–	●	–	–	–	●	–	–	●	–
U: CVD CREs	–	–	●	–	–	–	●	–	–	●	–
B: BoExp and xenobiotics CREs: CYP2A6 activity (*trans*-3'-hydroxycotinine, cotinine) and nicotine [h]	–	–	●	–	–	–	●	–	–	●	–
B: CVD CREs [i]	–	–	●	–	–	–	●	–	–	●	–
Pre-bronchodilator Spirometry testing [j] (also used for eligibility criteria at V1)	●	–	–	–	–	–	–	–	–	●	–
Post-bronchodilator spirometry testing [j]	●	–	●	–	–	–	●	–	–	●	–
Lung volume [j]	–	–	●	–	–	–	●	–	–	●	–
Cough questionnaire	–	–	●	–	–	–	●	–	–	●	–
FTND	●	–	–	–	–	–	–	–	–	●	–
MCEQ questionnaire	–	–	●	–	–	–	●	–	–	●	–
Product use diary (daily) [k]	–	●	●	●	●	●	●	●	●	●	–
Intent to use THS 2.2 questionnaire	–	–	–	●	–	–	–	–	–	●	–
Product preference question	–	–	–	●	–	–	–	–	–	–	–
Socio-economic status	–	–	●	–	–	–	–	–	–	–	–
AE/SAE recording [m]	●	●	●	●	●	●	●	●	●	●	●
U: Biobanking [l]	–	–	●	–	–	–	●	–	–	●	–
B: Biobanking for BoExp and CREs [l]	–	–	●	–	–	–	●	–	–	●	–
B: Biobanking for transcriptomics and lipidomics [l]	–	–	●	–	–	–	●	–	–	●	–

AE: Adverse event

B: Blood sample required

BMI: Body mass index
BoExp: Biomarkers of exposure
CC: Combustible cigarette(s)
CO: Carbon monoxide
COHb: Carboxyhemoglobin
CVD: Cardiovascular disease
CYP: Cytochrome P450 enzyme
ECG: Electrocardiogram
FTND: Fagerström test for nicotine dependence [36]
HIV: Human immunodeficiency virus
MCEQ: Modified cigarette evaluation questionnaire [33]
SAE: Serious adverse event
THS: Tobacco Heating System
U: Urine sample required
VAS: Visual analog scale

a. Subjects will be assessed for their motivation to quit by the means of Prochaska's questionnaire [37]. At V1, only subjects who are not willing to quit will be enrolled into the study to satisfy eligibility criteria.

b. Pre-study chest X-ray (with anterior-posterior and left lateral views) may be used if performed within six months prior to V1.

c. Safety laboratory parameters will be evaluated in at least 10 hours of fasting conditions (except at V1). **Hematology**: Hematocrit, hemoglobin, mean corpuscular hemoglobin, mean corpuscular hemoglobin concentration, mean corpuscular volume, platelet count, red blood cell count, white blood cell count, differential white blood cell count. Platelet count, white blood cell count from hematology will be evaluated as CREs. **Urine analysis**: pH, bilirubin, glucose, nitrite, red blood cell traces, protein specify gravity. **Clinical chemistry**: Albumin, total protein, alkaline phosphatase, alanine aminotransferase, aspartate aminotransferase, blood urea nitrogen, creatinine, gamma-glutamyl transferase, glucose, lactate dehydrogenase, potassium, sodium, total bilirubin, direct bilirubin, total cholesterol, triglycerides. Total WBC count will also be evaluated as part of the smokers' health profile.

d. Systolic and diastolic blood pressure, pulse rate, and respiratory rate. Vital signs will be assessed after resting at least five minutes in supine position.

e. Height (only at V1).

f. On V3, all enrolled subjects will be provided with THS 2.2 and will use THS 2.2 after the check-out from the clinic until V4. From V4, bi-monthly visit may be accommodated in order re-supply THS 2.2.

g. CO breath test at V3, V7 and V10: Once a day irrespective of product use in conjunction with COHb.

h. At V3, V7, and V10, one blood draw will be collected irrespective of the time of product use.

i. CREs for CVD: Blood draw will be taken after at least 10 hours of fasting conditions on V3, V7, and V10 (except for COHb).

j. At V1 and V10, the assessments must be performed in the following sequence:

- Pre-bronchodilator spirometry testing
- Pre-bronchodilator lung volumes using helium dilution (only at V10)
- Post-bronchodilator spirometry testing

At V3 and V7, the assessments must be performed in the following sequence:

- Pre-bronchodilator lung volumes using helium dilution
- Post-bronchodilator spirometry testing

k. Daily use of any tobacco/nicotine-containing products will be captured in the electronic diary from V2 onwards.

l. Once at V3, V7, and V10, samples will only be taken if additional consent for sample biobanking is given by the subject.

m. Spontaneous reporting of new AEs/SAEs by the subject and active follow up of ongoing AEs/SAEs by the site.
