# Supplementary material for: Evaluation of Biological and Functional Changes in Healthy Smokers Switching to the Tobacco Heating System 2.2 Versus Continued Tobacco Smoking: Protocol for a Randomized, Controlled, Multicenter Study
Source: JMIR Res Protoc. 2018 Aug 24;7(8):e11294. doi: 10.2196/11294 (PMC6128954; doi:10.2196/11294)
Supplement: Multimedia Appendix 2 [file resprot_v7i8e11294_app2.pdf]

Multimedia Appendix 2: Methods and Measurements

- The biomarkers of exposure to HPHCs selected for this study were among those the World Health Organization [44] has recommended be reduced in cigarette smoke and are derived from the FDA list of 18 HPHCs that are required to be reported for tobacco products.
- The HPHCs were specific to the source of exposure, with other sources being minor or nonexistent, reflecting a specific toxic exposure, or being a reliable surrogate of exposure, and exhibited an apparent half-life appropriate for the periods defined in this study. The list of HPHCs included a broad variety of chemical classes and organ toxicity classes, as defined by the International Agency for Research on Cancer (carcinogen, cardiovascular toxicant, respiratory toxicant, reproductive and development toxicant, addiction potential).
- The assays for biomarkers of exposure were validated as fit-for-purpose to meet the applicable portions of the FDA draft guidance. All biomarkers of exposure and the CREs 8-epi-PGF2α-III and 11-dehydrothromboxane B2 were analyzed by Celerion Laboratory.
- O-toluidine, 1-aminonaphthalene (1-NA), 2-aminonaphthalene (2-NA), 4-aminobiphenyl (4-ABP), S-phenylmercapturic acid (S-PMA), 1-hydroxypyrene (1-OHP), 3-hydroxybenzo(a)pyrene (3-B[a]P), total 4-(methylnitrosamino)-1-(3-pyridyl)-1-butanol (NNAL), and total N-nitrosonornicotine (NNN) were measured in urine after hydrolysis. Acidic hydrolysis was used for the measurement of the aromatic amines and S-PMA. 1-OHP, 3-B[a]P, total NNAL, and total NNN were hydrolyzed enzymatically.
- A direct analysis of urinary concentrations of monohydroxybutenyl mercapturic acid (MHBMA), 3-hydroxypropylmercapturic acid (3-HPMA), 3-hydroxy-1-methylpropylmercapturic acid (HMPMA), 2-cyanoethylmercapturic acid (CEMA), 8-epi-PGF2α-III, and 11-dehydrothromboxane B2 was performed by clinical sample

aliquots supplemented with internal standards and extracted either by a validated liquid-liquid or solid-phase extraction approach. The extracts were injected onto a qualified mass spectrometer or liquid chromatography mass spectrometer instrument and detected in multiple-reaction monitoring mode.

- Nicotine, cotinine and trans-3´-hydroxycotinine were measured in plasma using assays validated to meet the FDA guidance on Bioanalytical Method Validation [32]. Clinical samples were assayed after adding stable isotope-labeled internal standards to the aliquoted sample. Both assays used a solid-phase extraction to concentrate and purify the target analytes. The extracts were injected onto a qualified liquid chromatography tandem mass spectrometer instrument with positive ions detected in multiple-reaction monitoring mode.
- In addition to the total assays, nicotine equivalents were also measured with the direct analysis of nicotine, cotinine, trans-3′-hydroxycotinine, nicotine-N-glucuronide, cotinine-N-glucuronide, and trans-3′-hydroxycotinine-O-glucuronide in urine.
- Urinary creatinine and carboxyhemoglobin in whole blood were measured spectrophotometrically using assays validated by the College of American Pathologists/Clinical Laboratory Improvement Amendments.
- The CREs MPO, Platelet count, Fibrinogen, s-ICAM-1, Albumin, Homocysteine, hs-CRP, LDL Cholesterol, HDL Cholesterol, Triglycerides, Total cholesterol, Apo A1, Apo B, WBC, and Hemoglobin A1C will be measured using fit-to-purpose and validated methods by Covance Central Laboratory Services.
- Safety evaluations included vital signs, physical examinations, body weight, electrocardiography, safety laboratory tests (clinical chemistry, hematology, and urinalysis), and AEs/ SAEs. The intensity of AEs (graded as mild, moderate, or severe) and their relationship to the investigational products were also assessed. SAEs included, but were not limited to, medical events that resulted in death, were life-threatening, required inpatient hospitalization or prolongation of existing

hospitalization, resulted in persistent or significant disability/incapacity, or resulted from a congenital anomaly/birth defect.
